# Supplementary material for: The impact pathway of new quality productive forces on regional green technology innovation: A spatial mediation effect based on intellectual property protection
Source: PLoS One. 2025 Apr 8;20(4):e0319838. doi: 10.1371/journal.pone.0319838 (PMC11978015; doi:10.1371/journal.pone.0319838)
Supplement: Attachment — Submitted filename: Response to Reviewers.pdf [file pone.0319838.s003.pdf]

The Response to Reviewers for Major Revision

The Impact Pathway of New Quality Productive Forces on Regional Green Technology Innovation: A Spatial Mediation Effect Based on Intellectual Property Protection

To Chief Editor and Reviewer

We are grateful for the opportunity to submit a revised draft of the manuscript " The Impact Pathway of New Quality Productive Forces on Regional Green Technology Innovation: A Spatial Mediation Effect Based on Intellectual Property Protection" for publication in the PLOS ONE. We appreciate the time and effort that editors and the reviewers dedicated to providing feedback on our manuscript and are grateful for the insightful comments and valuable improvements to our paper. We have incorporated all of the suggestions made by the reviewers. Please see below, for a point-by-point response to the re- viewer' comments and concerns. All section numbers refer to the revised manuscript file with tracked changes.

Reviewer #1

The Reviewer's Comment #1.1

The title reflects the purpose of the research. The summary contains the purpose of the research, the data and methodology used and the main results. The introduction should be improved by giving an overview of the indicators' dynamics from 2011 to 2022. It is important to note below the tables what the values in brackets or *** represent for general public. The conclusions present the practical implications of the results of the empirical analysis.

Response:

Thank you very much for your nice reminder. We have fully revised and improved the manuscript in response to the suggestions. Specifically, we have added an overview of the dynamics of key indicators between 2011 and 2022 in the introduction section to better provide contextual support for the study, and added explanations of the specific meanings of parentheses and asterisks (***) underneath all the tables to enhance the readability of the data presentation. We hope that these adjustments will better meet the expectations of the reviewers and look forward to further feedback. The revision is in Section 1 [Ln51-63, 799-818].

"··· Over the past decade, China has witnessed significant dynamic changes in the development of green technology innovation and NQPF. Specifically, from 2011 to 2022, the number of green patent applications nationwide experienced steady growth, with the eastern developed regions demonstrating a marked advantage in both the quantity and quality of green technology innovation (Feng et al., 2022). Simultaneously, the NQPF index

exhibited an overall upward trend, albeit with notable regional disparities. The eastern coastal areas, leveraging robust economic foundations and technological advantages, achieved rapid advancements in NQPF, whereas the central and western regions showed comparatively slower progress. Furthermore, the intensity of intellectual property protection strengthened significantly during this period, particularly in the field of green technology patents, providing a solid institutional foundation for the dissemination and commercialization of innovation outcomes (Song et al., 2024). These dynamic changes not only reflect China's concerted efforts to promote green technology innovation but also highlight the persistent regional imbalances in development, thereby offering essential contextual support for exploring the relationship between NQPF and regional green technology innovation in this study. ⋯"

Reviewer #2

The Reviewer's Comment #2.1

Firstly, both the explanatory variable and the dependent variable should undergo Moran's index testing.

Response:

We are grateful to the reviewers for their suggestion to conduct Moran's index tests for both explanatory and dependent variables. We have added the test results of the global Moran's index for the explanatory variables in the revised manuscript and presented them in comparison in Table 4 to assess the spatial correlation of the variables. Once again, we thank the reviewers for their interest in the scientific validity and rigor of our study! The revision is in Section 4 [Ln371, 798-799].

The Reviewer's Comment #2.2

Secondly, please keep all regression coefficients to only 3 decimal places.

Response:

Thank you very much for your nice reminder. We have standardized all regression results in the revised manuscript to ensure that all regression coefficients and their corresponding standard errors are retained to three decimal places. This modification improves the consistency and standardization of the presentation of results, while facilitating a more intuitive understanding of the findings by the reader. We thank the reviewers for their attention to the precision of the presentation of our paper!

The Reviewer's Comment #2.3

Thirdly, in the third column of Table 5, the regression of spatial effects is not significant, and nQPF does not have spatial spillover effects on green technology innovation. Therefore, even if the quadratic term of nQPF is significant, it cannot prove its inverted "u" characteristic.

Response:

Thanks for your nice reminder. We have revisited the results and adjusted the interpretation. In the revised version, we further emphasize that the inverted "U" relationship of NQPF is mainly driven by local direct effects rather than through spatial spillovers. Although the spatial lagged effect (Wx) of NQPF is not significant in the third column, its quadratic term is significantly negative, suggesting that high levels of NQPF in neighboring regions may have a certain inhibitory effect on local green technology innovation. However, this inhibitory effect is not enough to support a significant spatial spillover effect, and thus cannot be taken as direct evidence of an inverted "U" shaped relationship. We have added a detailed explanation of this result in the Discussion section, while focusing our analysis on the contribution of local dynamics to the inverted "U" feature to avoid the possibility of over-inferring spatial spillover effects. We thank the reviewers for helping us to further improve the analytical logic and rigor of the paper! The revision is in Section 4 [Ln397-398,405-422].

"··· ~~This indicates that even when accounting for spatial dependence, nqpf still has a significant positive impact on RGTI.~~ ···"

"··· ~~The direct effects in column (4) show that NQPF are significantly positive at the 10% level, while their squared term is significantly negative at the 1% level. This indicates that the impact of nqpf on RGTI follows a notable inverted U-shaped pattern, displaying non-linear characteristics. This occurs because the direct effects may include feedback from the green technological innovation capabilities of surrounding regions, which could suppress the local innovation capacity. In the early stages of nqpf development, it positively promotes green technological innovation, encouraging enterprises and regions to engage in more green technology research and development activities. However, as nqpf reaches higher levels, the marginal benefits of innovation begin to decline, and excessive resource input and concentration of productive forces may, in fact, inhibit further innovation.~~ However, the Wx effect of NQPF in Column (4) is not significant (1.857, $p > 0.1$), indicating that the direct spatial spillover effect is limited. The quadratic term of NQPF in the Wx effect is significantly negative (-23.511, $p < 0.01$), suggesting that high levels of NQPF in neighboring regions may inhibit local innovation. This could potentially result from resource competition or path dependency in innovation activities. As indicated by the significant direct effects in Column (4), the inverted "U" relationship of NQPF is primarily driven by local dynamics. Although the spatial lag effect of NQPF

itself is not significant, the significantly negative quadratic term suggests that high levels of NQPF in neighboring regions may hinder local green innovation. This phenomenon may stem from inter-regional competition for innovation resources or a crowding-out effect caused by the excessive concentration of high-level productive forces. ···"

The Reviewer's Comment #2.4

Fourthly, the literature review and hypothesis proposal sections need to be revised to better introduce the mediating variable IPP.

Response:

Thank you very much for your nice reminder. We have comprehensively revised and supplemented the relevant contents in the revised draft. In the section of Literature Review and Theoretical Hypotheses, we have added a systematic discussion on intellectual property protection as a mediating variable, and elaborated in detail on the mechanism of its role in promoting green technological innovation, including its facilitating effect on technology diffusion, its incentive effect on the protection of innovation achievements, and its key impact on the regional innovation ecological optimization, in order to more clearly illustrate its role as a bridge between new quality productivity and regional green technology innovation. We thank the reviewers for helping us to improve the theoretical framework and logical system of the paper! The revision is in Section 2 [Ln131-143].

"··· Firstly, new quality productive forces (NQPF) accelerate technological innovation and industrial upgrading, introducing more complex demands for intellectual property protection. This drives the expansion of protection coverage and the refinement of institutional frameworks (Guangqin and L, 2024). For instance, with the advancement of green and digital technologies, emerging innovations require more comprehensive protection mechanisms to address the challenges posed by rapid technological iteration and cross-sector integration (Jie and Li, 2024). Secondly, the enhancement of NQPF intensifies inter-regional technological competition, increasing the reliance on robust intellectual property protection to ensure the equitable distribution of innovation outcomes and the controlled diffusion of technologies (Luo et al., 2024). Furthermore, the diversified characteristics of NQPF, such as digitalization and greening, impose reform demands on traditional intellectual property protection systems, driving continuous improvements in legal frameworks and enforcement mechanisms (Lu et al., 2024). Therefore, NQPF serves not only as a critical driving force for intellectual property protection reform but also as a fundamental pillar in enhancing regional and national competitiveness. ···"

The Reviewer's Comment #2.5

Fifth, the variables in the article need to be explained morecarefully, including definitions, sources, and reasons for selecting control variables.

Response:

Thanks for your nice reminder. In the Theoretical Frameworks and Hypotheses section, we expand upon the innovation-driven theory and the Ability-Motivation-Opportunity theory. The innovation-driven theory is discussed in detail, highlighting its macro perspective on the effects of implementing IPP systems on RTI within national research project teams. Additionally, we explore RTI's influences from an intra-team viewpoint, employing the micro perspective offered by AMO theory, which is elaborated to analyze team dynamics. The revision is in Section 2 [91-143,199-207].

"··· RGTI refers to innovative activities that promote the efficient utilization of resources, pollution reduction and ecological protection through the research and development, application and promotion of green technologies in a specific region with the goal of sustainable development (Hao et al., 2024). ···"

"··· NQPF refers to a new form of productivity in the modern economic system with knowledge, technology, green development and digitalization as the core drivers (Guangqin and L, 2024). ···"

"··· ~~Given the numerous factors that influence RGTI, this study incorporates several control variables into the model to minimize their potential confounding effects: (1) Level of Economic Development (lnpgdp): Cities with higher levels of economic development are better equipped to provide the necessary financial resources for regional green innovation. (2) Urbanization Rate (ur): The agglomeration effects of urbanization attract human and physical capital from surrounding areas, accelerating regional innovation capacity. (3) Industrial Structure (ind): Industrial clusters can generate economies of scale and promote regional technological advancement. However, the concentration of manufacturing and producer services may also exacerbate competition between firms, potentially lowering production efficiency. (4) Energy Consumption Structure (ecs): The energy consumption structure reflects the actual application of clean energy and technology in each region, influencing the development of green innovation. (5) Technological Investment (teci): Government intervention in technological research and development, as well as in the market economy, will inevitably affect the efficiency of regional green innovation. (6) Degree of Openness (op): Openness to external markets attracts foreign enterprises with advanced innovation technologies to engage in green economic activities, impacting the efficiency of regional green innovation. (7) Environmental Regulation Intensity (eri): Environmental regulations can directly influence the efficiency of regional green innovation by reducing environmental pollution. (8) Human Capital (hr): Human capital is a key factor in economic development. Beyond influencing financial agglomeration, it also affects regional green innovation~~

~~efficiency by enhancing firms' technical expertise and innovation capabilities.~~ To account for the various factors influencing regional green technology innovation (RGTI), this study incorporates several control variables into the model to ensure the robustness and reliability of the empirical analysis. First, the level of economic development, measured as the logarithm of per capita GDP, reflects a region’s financial and infrastructural capacity to support green innovation. Second, the urbanization rate, defined as the proportion of the urban population to the total population, captures agglomeration effects that attract talent and resources, thereby enhancing regional innovation capacity. Third, the industrial structure, represented by the proportion of the tertiary sector in regional GDP, indicates industrial advancement, which can facilitate technological innovation and diffusion. Fourth, the energy consumption structure, measured as the proportion of coal consumption in total energy consumption, reflects the region’s reliance on clean energy and its application of green technologies. Fifth, technological investment, calculated as the proportion of local government fiscal expenditure on science and technology, represents government support for innovation, which directly affects green innovation outcomes. Sixth, the intensity of environmental regulation, proxied by the proportion of industrial pollution control investments in industrial added value, incentivizes firms to adopt green technologies and improve innovation efficiency. Finally, human capital, expressed as the number of full-time teachers in higher education institutions per 10,000 people, captures the region’s technical expertise and innovation capacity, both critical for driving green innovation. These variables, sourced from national and regional statistical yearbooks, provide a comprehensive framework to control for the confounding effects of regional heterogeneity in the analysis.. …"

"… IPP refers to a series of systems and measures to provide legal protection for intellectual achievements and identifying resources created by individuals or organizations in the scientific, technological, cultural and commercial fields through legal, administrative and technical means (Abdin et al., 2024). …"
